# The DC-SIGN-CD56 interaction inhibits the anti-dendritic cell cytotoxicity of CD56 expressing cells

**DOI:** 10.1186/s13027-015-0043-8

**Published:** 2015-12-10

**Authors:** Alexey A. Nabatov, Ivan S. Raginov

**Affiliations:** Science Center, Volga Region State Academy of Physical Culture, Sport and Tourism, 33, Universiade Village, Kazan, 420138 Russia; Department of Molecular Cell Biology & Immunology, VU University Medical Center, Amsterdam, The Netherlands; Republican Clinical Hospital, 138 Orenburgsky tract, Kazan, 420064 RUSSIA; Scientific and Educational Center of Pharmaceutics, 18 Kremlyovskaya ul., Kazan, 423000 RUSSIA

**Keywords:** CD56/NCAM, Natural killer cells, HIV-1 infection, DC-SIGN, Dendritic cells, Cytotoxicity, Polysialic acid

## Abstract

**Background:**

This study aimed to clarify interactions of the pattern-recognition receptor DC-SIGN with cells from the HIV-infected peripheral blood lymphocyte cultures.

**Methods:**

Cells from control and HIV-infected peripheral blood lymphocyte cultures were tested for the surface expression of DC-SIGN ligands. The DC-SIGN ligand expressing cells were analyzed for the role of DC-SIGN-ligand interaction in their functionality.

**Results:**

In the vast majority of experiments HIV-infected lymphocytes did not express detectable DC-SIGN ligands on their cell surfaces. In contrast, non-infected cells, carrying NK-specific marker CD56, expressed cell surface DC-SIGN ligands. The weakly polysialylated CD56 was identified as a novel DC-SIGN ligand. The treatment of DC-SIGN expressing dendritic cells with anti-DC-SIGN antibodies increased the anti-dendritic cell cytotoxicity of CD56^pos^ cells. The treatment of CD56^pos^ cells with a peptide, blocking the weakly polysialylated CD56-specifc *trans*-homophilic interactions, inhibited their anti-dendritic cells cytotoxicity.

**Conclusions:**

The interaction between DC-SIGN and CD56 inhibits homotypic intercellular interactions of CD56^pos^ cells and protects DC-SIGN expressing dendritic cells against CD56^pos^ cell-mediated cytotoxicity. This finding can have an impact on the development of approaches to HIV infection and cancer therapy as well as in transplantation medicine.

## Background

Immune tolerance, a state of unresponsiveness of the immune system to specific immunogens, can be reached by different mechanisms, including the dysregulation of dendritic cells (DCs). As antigen presenting cells, DCs play a central role in effective immune response development. Complete dysregulation or depletion of DCs causes immunodeficiency. Although different stages of HIV and SIV infections vary in the level of DC dysregulation, the depletion of DCs from blood stream and from lymph nodes is characteristic for late stages of these infections [1–4]. The DC functional deficiency can be the key element for AIDS development. However, the mechanisms causing the HIV infection-associated DC depletion remain vague. Among other possible factors natural killer (NK) cells might mediate the DC depletion in HIV infection similar to their participation in the CD4+ T cell depletion [5]. HIV infection is associated with the dramatic depletion of DCs in lymph nodes, the main place of antigen presentation by the mature DCs (mDCs), expressing full range of MHC class I and II molecules [6]. At the same time, SIV infection causes accumulation of cytotoxic CD16+ NK cells (analogues of CD56^dim^CD16^pos^ ones in human) in lymph nodes where they are not normally present, in contrast to the much less cytotoxic cytokine secreting CD56^hi^CD16^neg^ NK cells that are normal for lymph nodes [7–9].

DC-SIGN (Dendritic Cell-Specific Intercellular adhesion molecule-3-Grabbing Non-integrin; CD209) is a C-type lectin receptor present on macrophages and dendritic cells. DC-SIGN interacts with high-mannose oligosaccharides conjugated to HIV-1 envelope glycoprotein gp120. This interaction can initiate the temporal internalization of HIV virions and increase the efficacy of HIV transmission [10]. HIV-1 infected cells can express gp120 on their cell surfaces where gp120 can be recognized by DC-SIGN-expressing dendritic cells. The ability of DC-SIGN expressing cells to enhance HIV infection was originally found for cells from placenta [11]. These cells appeared to be DCs forming tight interaction with natural killer (NK) cells [12–14]. In normal decidua, DC-SIGN^pos^ immature dendritic cells (iDCs) form one of the most presented cell populations whereas spontaneous abortions are associated with the decreased number of these cells [15, 16]. As a part of pregnancy immune tolerance mechanisms decidual NKs seem to inhibit iDCs maturation and prevent mature DCs from migrating to the lymphoid tissues [17, 18]. The killing of the maturing DCs in placenta occurs despite the maturation-associated growth of the cell surface density of major histocompatibility complex (MHC) class I, a known factor of NK cytotoxicity inhibition. On the other hand, the maturing DCs moderately decrease the surface expression of DC-SIGN [19]. The role of DC-SIGN and its ligands (including the HIV associated ones) in the inhibition of NK cytotoxicity remains unclear.

In this study interactions of DC-SIGN with HIV-infected peripheral blood lymphocytes (PBLs) were analyzed. The cell surface DC-SIGN ligand (DC-SIGN-L) expression was found as a rare event for HIV-1 infected CD4 lymphocytes. In contrast, non-infected CD56^pos^ cells were mostly found as DC-SIGN-L^pos^ ones. The weakly polysialylated CD56 was identified as a novel ligand for DC-SIGN that can regulate anti-DC cytotoxicity of CD56^pos^ cells. These findings give a better understanding of HIV-infection pathogenesis and pregnancy-associated immune tolerance.

## Results

### DC-SIGN rarely binds to HIV-1 infected cells

**Fig. 1:**
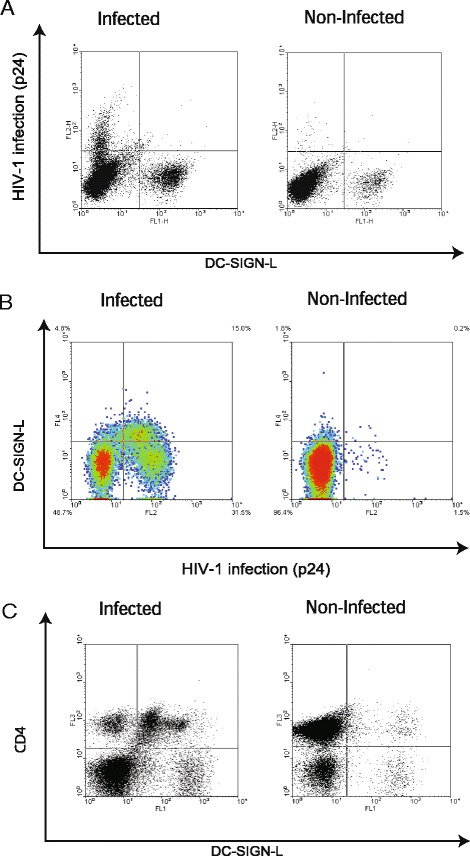
DC-SIGN binding to cells in HIV-1 infected cultures. **a** The typical dotplot of HIV-1-infected and non-infected control and PBLs stained for DC-SIGN-L and HIV-1 core protein p24. **b** The density plot of rare HIV-1 infected and non-infected PBLs stained as described above in Fig. **a**. **c** The dotplot of HIV-1 infected and non-infected PBL cultures stained for CD4 and DC-SIGN-L

To study the interactions between HIV-1 infected cells and DC-SIGN, donor PBLs were activated, infected with HIV-1 and stained with a soluble DC-SIGN-Fc chimera. Infected PBLs were expected to bind soluble DC-SIGN due to the cell surface expression of HIV-1 envelope glycoprotein gp120. However, in the vast majority of experiments HIV-1 infected (viral core protein p24 positive (p24^pos^)) cells did not express DC-SIGN-L (Fig. 1a). Nevertheless, for some donors the p24^high^DC-SIGN-L^neg^ and p24^low^DC-SIGN-L^dim^ cell populations were found (Fig. 1b). When the rare donors infected cells were stained for CD4, an additional population of DC-SIGN-L^pos^CD4^pos^ cells was detected (Fig. 1c). Thus, the used culturing conditions either stimulate the gp120 shedding from the surface of infected cells or the HIV budding occurs inside cells and gp120 is simply not present on the infected cell surface. The accumulation of p24 in the p24^high^DC-SIGN-L^neg^ cells supports rather the intracellular budding option.

Surprisingly, a population of non-infected cells gave a high signal specific for DC-SIGN-L (Fig. 1a and c). From this point the focus of research shifted away from HIV-1 infected cells to the characterization of this p24^neg^DC-SIGN-L^pos^ population.

### DC-SIGN binds to CD56+ cells

**Fig. 2:**
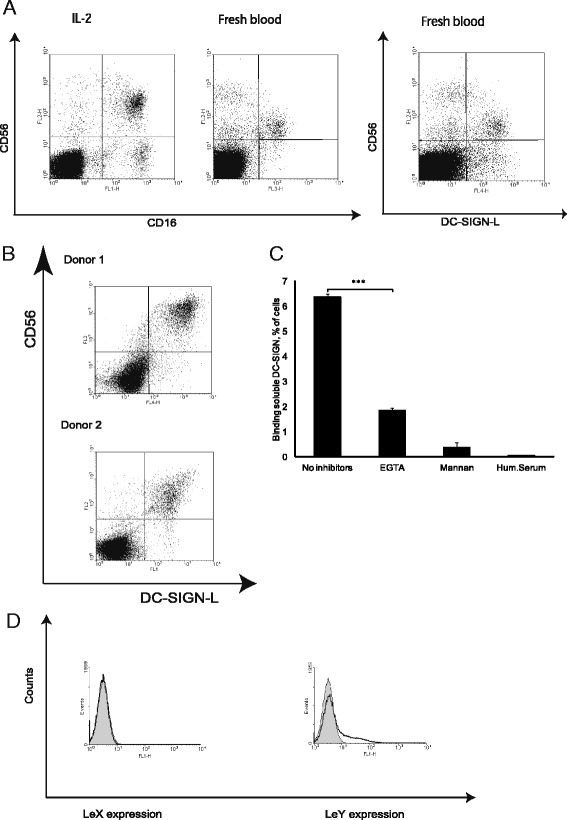
DC-SIGN-Fc binds in a C-type lectin-specific manner to CD56^pos^ cells expressing Lewis Y carbohydrates. **a** The dot plots of activated and freshly isolated PBLs stained for DC-SIGN-L, CD56 and CD16. **b** The dot plots of activated PBLs stained for CD56 and DC-SIGN-L. **c** The DC-SIGN-Fc binding to CD56^pos^ cells was analyzed in the absence and in the presence of DC-SIGN inhibitors: mannan (2 mg/ml), EGTA (10 mM). Human serum (Hum. Serum) was used as a negative control for unspecific human Fc fragment bindings. The experiment was performed in triplicates and error bars represent ± SD of triplicates. **d** Histograms of CD56^pos^ (clear plots) and CD56^neg^ (grey plots) activated PBLs stained for -Lewis X (left) and Lewis Y (right) carbohydrates surface expression with specific antibodies

The phenotypic analysis identified the p24^neg^DC-SIGN-L^pos^ cells as CD3+/−CD56 + CD11b + CD16 + CCR5-CXCR4-CD161+/−CD57+/–ones. The CD56 or NCAM (Neural Cell Adhesion Molecule; an NK-and neuron-specific antigen) cell surface expression suggests that these cells belong to a heterogenic population of NK and NKT cells (NK (T) cells) that according to the phenotype and culturing conditions should have some features of non-MHC-restricted cytokine induced killers [20, 21]. In human blood, two main subsets of NK cells have been described, cytolytic CD56^dim^CD16^pos^ and non-cytolytic cytokine secreting CD56^bright (hi)^CD16^neg^ subsets, of which the CD56^dim^CD16^pos^ subset predominates [9]. CD56^hi^CD16^neg^ subset did not bind DC-SIGN (Fig. 2a). This result can reflect the known difference in the level of CD56 polysialylation found for cytotoxic and non-cytotoxic NK subsets [22]. Notably, the activation of PBLs caused depletion of the CD56^hi^CD16^neg^ NK subset and increased the CD56 surface expression that clearly correlated with the level of DC-SIGN-L expression (Fig. 2a, b). The interaction between the soluble DC-SIGN and CD56^pos^ cells was lectin specific, since it could be inhibited by the polycarbohydrate mannan and the calcium-chelator EGTA (Fig. 2c).

Lewis (Le) carbohydrates are specific DC-SIGN ligands [23]. The Le^X^ (Gal1-b-4 [Fuc1-α-3] GlcNAc)-containing structures (Le^X^ or Le^Y^) have also been found on NCAM [24]. To clarify the expression of Le^X^-like structures, the activated PBLs were stained with antibodies to CD56, Le^Y^ and Le^X^ carbohydrates. Both CD56^pos^ and CD56^neg^ cells showed no difference in the level of Le^X^ expression. However, CD56^pos^ cells had the higher Le^Y^ expression (Fig. 2d).

### Weakly-polysialylated CD56 is a novel DC-SIGN ligand

**Fig. 3:**
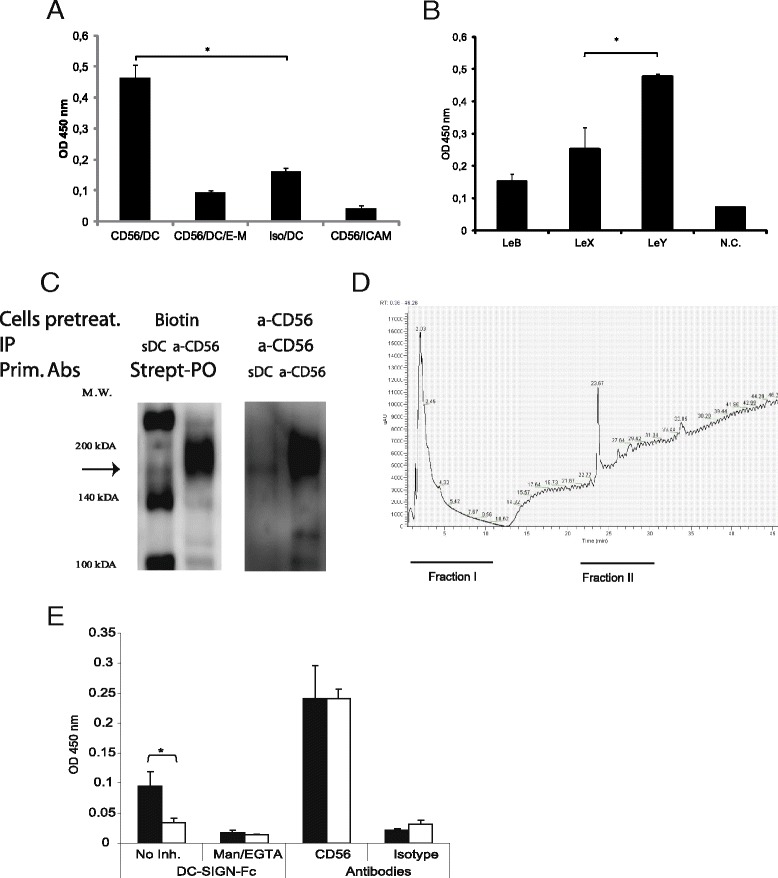
Identification of non-polysialilated CD56 as a novel ligand of DC-SIGN. **a** The presorbed anti-CD56 or isotype (Iso) control antibodies were incubated with PBL lysates. DC-SIGN-Fc (DC) binding to the captured CD56 alone or in the presence of inhibitors (EGTA and mannan, E-M) was assessed. ICAM-3-Fc (ICAM) was used as a negative control. **b** The binding of anti-Lewis sugars antibodies (Le) to the captured CD56 was assessed. Negative control (N.C.) was performed without anti-Lewis antibodies. **a** and **b** experiments were performed in triplicates and error bars represent ± SD of triplicates. **c** The immunoprecipitated DC-SIGN ligands (sDC) and CD56 (a-CD56) from activated PBLs, pretreated with biotin, were analyzed in immunoblot with streptavidin, left panel. The immunoprecipitated cell surface CD56 was analyzed in immunoblot with DC-SIGN-Fc and anti-CD56 antibodies, right panel. The arrow points out a common for the two immunoblots band. **d** Results of anion exchange (AEX) chromatography separation of immunoprecipitated CD56 from the activated PBL lysate. Fractions I and II were collected. **e** The fraction I (black bars) and fraction II (white bars) obtained in the AEX experiment were directly sorbed of ELISA plates and analyzed for binding anti-CD56 antibodies and DC-SIGN-Fc with or without of inhibiting mannan/EGTA (Man/EGTA) mixture

The obtained data strongly suggest that CD56^pos^ activated PBLs express a DC-SIGN ligand on their cell surface. To clarify mechanisms mediating the correlation between the levels of CD56 and DC-SIGN-L expression (Fig. 2b), CD56 from cell-lysates was captured with the presorbed specific antibodies and the interaction with soluble DC-SIGN was determined in ELISA. The captured CD56 interacted with soluble DC-SIGN in a lectin-specific manner (Fig. 3a). The similar approach showed that CD56 is associated with Le^Y^ carbohydrates (Fig. 3b).

To further clarify relationships between DC-SIGN and CD56, the analysis of cell surface-associated CD56 and DC-SIGN-L was performed using immuneprecipitation followed by immunoblot. The immunoprecipitated DC-SIGN ligands and CD56 from the cells, pretreated with biotin, were analyzed in immunoblot with streptavidin (Fig. 3c, left panel). The immunoprecipitated cell surface CD56 was analyzed in immunoblot with DC-SIGN-Fc and anti-CD56 antibodies (Fig. 3c, right panel). The results of the immunoblots indicate that a 180 kDa DC-SIGN ligand is present both in the lane of cell surface proteins and in the lane of immunoprecipitated CD56 (Fig. 3c). In latter case the DC-SIGN-specific signal corresponds to the CD56-180 isoform of CD56, having several isoforms different in their protein molecular weight [25]. The CD56 specific signal in both immunoblots revealed a smeared band corresponding to 175–200 kDa characteristic for CD56 (Fig. 3c) [25]. This 175–200 kDa band is known to reflect the length difference of negatively charged polysialic acid (PSA) conjugated to the 180 kDa protein backbone of CD56 [25]. The staining with anti-polysialic acid confirmed the PSA-related nature of the smeary CD56 band (data not shown). These results confirm that the cell surface CD56-180 is a pool of 180 kDa protein molecules that differ in the length of the attached PSAs, forming the wide smeared band [25]. However, the results also specify that DC-SIGN interacts on the cell surface with the non-or slightly polysialylated variants of CD56-180, forming a narrow band in the area corresponding to 180 kDa protein on immunoblot.

The following anion-exchange HPLC of CD56 from cell surface showed the presence of two main protein containing fractions different in their column binding properties (Fig. 3d). The retention of fraction II on the column suggests that compounds from this fraction are highly negatively charged in contrast to the fraction I that moved through the column without retention. CD56 was detected equally by ELISA in both fractions but the DC-SIGN lectin-specific interaction with fraction I was significantly higher (Fig. 3e), which supports the hypothesis that DC-SIGN interacts with the weakly polysialylated form of CD56.

### DC-SIGN affects the anti-DC cytotoxicity of CD56^pos^cells that depends on weakly polysialylated-CD56 *trans*-homophilic interactions

**Fig. 4:**
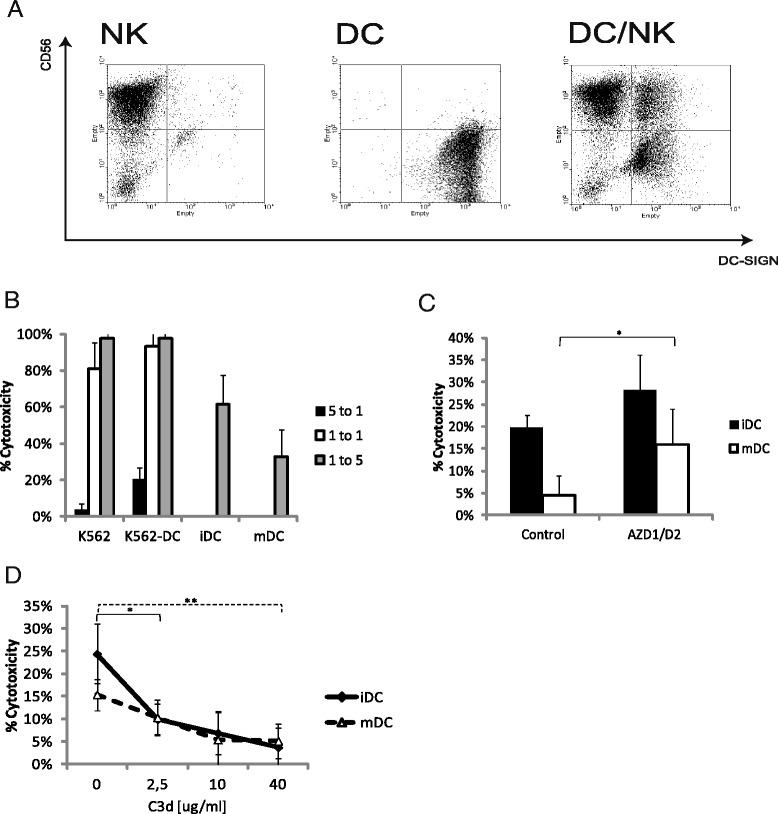
NK–mediated cytotoxicity of DC but not K562 depends on both DC-SIGN and CD56 trans-homophilic interaction. **a** The dotplots of isolated CD56^pos^ cells (NK), DC-SIGN expressing DCs and their co-culture. Preincubation of DCs with anti-DC-SIGN antibodies or mannan did not change the picture; **b** The CD56^pos^ cell cytotoxicity of K562, DC-SIGN-expressing K562, iDCs and mDCs taken in different effector/target ratios. **c** Pre-incubation of DCs with DC-SIGN lectin inhibiting antibodies (AZD1/D2) increases the CD56 cytotoxicity of DC. Error bars represent ± SD of experiment quadruplicates. **d** The *trans*-homophilic interaction blocking peptide C3d inhibits the CD56^pos^ cell cytotoxicity of DCs. Error bars represent ± SD of experiment quadruplicates. In experiments **c** and **d** ratio DC:NK was 1:8. The results are representative from several experiments

The functional DC-SIGN–CD56 interaction suggests a tight interaction between DCs and NK cells. Indeed DCs and the CD56^pos^ cells formed tight cellular complexes. Preincubation of dendritic cells with anti-DC-SIGN antibodies or mannan did not change the complex formation (Fig. 4a). This suggests that the interaction of CD56 with DC-SIGN rather regulates cell activities than plays a substantial role in adhesion between NK and dendritic cells, mediated by multiple other adhesion molecules.

NK cells are known to kill cells that do not express MHC class I on their surface and the mechanism of this killing is well described. NK cells also can kill MHC class I expressing cells but the mechanism of this killing remains unclear. CD56 does not play a role in the NK cell mediated lysis of K562 cells, a model target cell line expressing no MHC class I [26]. In the light of the described above results this suggests that DC-SIGN also does not play a substantial role in the killing of K562 cells. To clarify the role of DC-SIGN for K562 cells and DCs cytotoxic death, CD56^pos^ cells were used in the cytotoxicity test with target K562 cells and DCs in different ratios of target/effector (T/E) cells. The killing of K562 cells was detected even at T/E ratio of 5 to 1. The DC-SIGN expression did not significantly (with a trend to increase for DC-SIGN-K562 cells) change the lysis of K562 cells (Fig. 4b). However the killing of DCs by the same CD56^pos^ cells was detected only at 1 to 5 T/E ratio. These results strongly suggest deeply different mechanisms of the K562 and DCs killing where the killing of DCs needs a certain cooperation of CD56^pos^ cells. To further clarify the role of DC-SIGN in the CD56^pos^ cell cytotoxicity of DCs, the latter, pretreated with anti-DC-SIGN antibodies, were used in the cytotoxicity experiments. These experiments revealed that the inhibition of DC-SIGN lectin activity by antibodies significantly increased the killing of mDCs (Fig. 4c).

The non-polysialylated (non-PSA) NCAM (CD56) forms both *cis*-and *trans*-homophilic (CD56-CD56) interactions whereas homotypic intercellular interactions between NK cells are required for certain types of NK cytotoxicity [27–29]. However, the functionality of CD56 surface expression on NK cells remains unclear. To investigate the role of the CD56-mediated homophilic interactions in the anti-DC cytotoxicity of CD56^pos^ cells, the C3d dendromeric peptide, blocking the *trans*-homophilic NCAM interaction, was used [30]. Pre-incubation of CD56^pos^ cells with the C3d peptide significantly decreased the killing of both iDC and mDCs in a dose-dependent manner (Fig. 4d).

Altogether these results suggest that CD56 and DC-SIGN do not play a significant role in the MHC class I negative target cell killing. However, these molecules seem to play very important role in the fate of DCs interacting with CD56^pos^ cells.

## Discussion

HIV-1 infection is characterized by the high level of immune activation. The current regimens of antiretroviral therapy are unable to inhibit this antigen presenting cell-driven viral-induced immune hyperactivation [31]. On the other hand, the presence of host factors that inhibit this immune activation can be assumed. The immune activation seems to mediate the optimal conditions for HIV propagation in host. This has its reflection in the activation of non-infected donor PBLs as a preparation step for HIV infection *in vitro*. The protocol of PBLs activation for HIV infection resembles the one for induction of cytokine induced killer cells, used in immune therapy of tumors [21]. Therefore, it was not a surprise that on the day 10–14 of HIV-1 infection in the presence of IL-2 a substantial number of cells, carrying specific NK marker CD56, was detected. The surprise was that those CD56^pos^ were often the main binders of soluble DC-SIGN, used originally to detect the surface expression of HIV-1 envelop gp120 on infected PBLs. The rare finding of DC-SIGN-L^pos^ cells among CD4 HIV-infected PBLs reflects rather the rare budding HIV-1 virions from the cell surface. The finding of DC-SIGN-L^neg^p24^high^ infected cells in the majority of the studied donor lymphocytes suggests accumulation of viral particles inside the infected cells before their release. This supports the findings that HIV virions are mostly released using exocytosis from intracellular multivesicular bodies wherein they bud [32]. This strategy allows virus to prevent the recognition of infected cells by immune system.

Non- or slightly polysialylated CD56 was found as a novel DC-SIGN ligand on the surface of activated CD56^pos^ cells. NCAM is known to be present in polysialylated and non-polysialylated forms strikingly different in their functions [27]. Among the potential DC-SIGN ligands the Le^Y^ oligosaccharides were identified as associated with CD56, which confirms the described presence of NCAM-conjugated Le^X^–like carbohydrates [24]. The short or medium-length PSA chains on CD56 are characteristic for active cytotoxic NK cells whereas cytotoxically inactive NK cells express long PSA, suggesting different glycosylation machinery in these two NK populations [22]. The obtained phenotyping data suggest that the found CD56^pos^DC-SIGN-L^pos^ cells resemble the cytotoxic CD56^dim^CD16^pos^ subset of NK cells whereas non-cytotoxic CD56^hi^CD16^neg^ cells, more characteristic for lymph nodes, do not bind DC-SIGN. Thus, DC-SIGN–CD56 interaction plays a role mostly for cytotoxic NK (T) cells. Of interest, in the presence of the transforming growth factor-β and IL-15 (normal in human decidua) CD56^dim^CD16^pos^ NK cells convert into CD56^dim^CD16^neg^ NK cells with properties similar to decidual NK cells [33].

The inhibition of DC-SIGN lectin activity with specific antibodies increased the anti-DC cytotoxicity of CD56^pos^ cells, which suggests a DC-SIGN inhibitory role in the regulation of the CD56^pos^ cells cytotoxicity when these cells tightly interact with DCs. On the other hand, the absence of the DC-SIGN protective effect for K562 cells, having no MHC class I expression, suggests that the DC-SIGN-mediated protective effect is significant mostly for MHC class I expressing target cells. These data are in line with the findings that CD56 on NK cells does not mediate the killing of K562 cells but the killing of more complex, in terms of MHC expression, targets [26].

The polysialylated molecules are highly negatively charged and electrostatically repel each other on the same and neighbor cells. In contrast to polysialylated CD56, the tips of non-or slightly polysialylated CD56 molecules form bonds with CD56 on the same and neighboring neurons (*cis*-and *trans*-homophilic interactions, respectively). The *cis*-homophilic bonds create a kind of cell surface shield that can be used for intercellular contacts and protection of underlying structures (including the NCAM conjugated Le^X^-like structures) against unspecific bindings [24, 27]. This can explain why anti-LeY antibodies, in contrast to soluble DC-SIGN, interacted with a relatively small part of CD56^pos^ cells from the activated PBL cultures.

NK cytotoxicity is highly controlled by intercellular interactions including homotypic (NK-NK) ones [28, 29, 34]. The inhibition of anti-DC cytotoxicity of CD56^pos^ cells in the presence of the C3d peptide, inhibiting *trans*-homophilic CD56 interactions, suggests the functional role of CD56 homophilic bonds in the MHC class I independent CD56^pos^ cytotoxicity. The DC-SIGN-CD56 interaction sterically excludes the interacting CD56 molecule from other interactions including the *trans*-homophilic ones mediating cytotoxicity, which decreases anti-DC cytotoxicity of CD56^pos^ cells.

The mimicry, simulating the normal host structures or functions, is a well-known survival strategy of microbial pathogens that allows them to avoid or to suppress the host immune system response and co-exist with the host. The immunoglobulin-like receptor CD56 is heavily N-glycosylated, has the five loops protein structure and can bind DC-SIGN, which strikingly resembles the structure and properties of HIV-1 envelope gp120. Thus, the host immune system might take the HIV infected cells expressing envelope gp120 for the activated CD56^pos^ cells. Moreover, shedded gp120 might inhibit DC-SIGN interactions with CD56 and induce DC depletion.

## Conclusions

This study demonstrates that the DC-SIGN expression is one of the DC maturation state factors that can define the level of NK (T) cell cooperation-dependent cytotoxicity. These findings suggest DC-SIGN ligands as new immune tolerogenic compounds that might be applied for the NK (T)-mediated regulation of both the number of DCs and the level of DCs maturation. The used in this study approaches on the inhibition of CD56-dependent cytotoxicity allow targeting MHC class I independent cytotoxicity of NK (T) cells. Altogether these results can be useful in development of new approaches to the non-MHC-restricted cytokine induced killer cells-based therapies, the maintenance of pregnancy, transplant rejection inhibition and HIV infection treatment.

## Materials and methods

### Antibodies

The following monoclonal antibodies were used: AZN-D1 and-D2 (DC-SIGN) [12], UCHT1 (CD3), 3G8 (CD16), L307.4 (CD80), 2331 (CD86), G46-6 (HLA-DR), B159 (mouse IgG1) and NCAM16.2 (mouse Ig2b) (both CD56), MOPC-21 (IgG1 isotype control), NK-1 (CD57) (all from BD Pharmingen). Mouse mAbs against Lewis sugar structures: Lewis X; Lewis Y, and Lewis B were purchased from Calbiochem. Anti-PSA monoclonal antibody (735) was a generous gift from Dr. Martina Muhlenhoff.

The used soluble DC-SIGN-Fc chimera consisted of the extracellular portion of DC-SIGN (aa residues 64–404) fused at the C terminus to the human IgG1-Fc domain [35]. DC-SIGN-Fc was produced in Chinese hamster ovary K1 cells after transfection with the DC-SIGN-Sig-pIgG1-Fc vector (20 μg). ICAM-3-Fc that contains the same as Fc fragment as DC-SIGN-Fc or 10 % human sera were used as a negative control for Fc binding.

The fluorochrome-conjugated monoclonal antibodies used in flow cytometry were: CD3 (BD-PharMingen), CD11b (Coulter); DC-SIGN (R&D Systems) CD3, CD4, CD8, CD56 (all from Beckman Coulter), biotinylated anti-CD16 CD86, HLA-DR (Both from BD-PharMingen); PerCP labeled anti-human CD4; anti-mouse IgG and goat anti-human Fc antibodies (Jackson Immunoresearch Lab. Inc.).

### Cells

The NK-susceptible K562 (human chronic myelogenous leukemia) cell line, which lacks HLA class I expression, as well as K562 expressing DC-SIGN (K562-DC) [12] were used as a positive control target in cytotoxicity assays. Ficoll isolated PBL were activated with 5 μg/ml phytohemagglutinin (Sigma) and cultured in RPMI medium containing 10 % fetal calf serum, penicillin (100 units/ml), and streptomycin (100 units/ml) with recombinant interleukin-2 (100 units/ml) (Strathmann Biotech Ag) for 3 days. On day 4 PBLs were washed and cultured in the same culture medium but without PHA. For HIV infection experiments HIV-1 (JRCSF) (100 TCID_50_ per 10^5^ cells) was added to the PBL culture on day 1 after the PHA stimulation. CD56^pos^cells were isolated with anti-human CD56 microbeads (Miltenyi Biotec GmbH) from the 10–14 days old PBL cultures. Isolated NK cells were routinely tested for CD56 surface expression. The purified CD56^pos^ cells were cultured in the cell free PBL medium culture for 24 h before used in the experiments.

Immature and mature DCs were derived from monocytes as described [36]. Briefly, monocytes were isolated from buffy coats of healthy donors by Ficoll centrifugation and MACS sorting for CD14^+^ cells using CD14 microbeads (Miltenyi Biotec GmbH). Isolated monocytes were cultured for 7 days on RPMI 1640 10 % FCS in the presence of IL-4 (600 U/ml) and GM-CSF (800 U/ml) to obtain immature DCs and in the presence of LPS (from *Escherichia coli*, Sigma-Aldrich, St Louis, MO, 2 μg/ml) in the final 2 days to obtain mature DCs. Maturation of DCs was followed using cell staining for the maturation markers as CD86 and HLA-DR.

### DC-SIGN-Fc cell staining

DC-SIGN-Fc cell staining was performed to find expression of DC-SIGN ligands on the cell surfaces. PBLs were incubated with DC-SIGN-Fc for 30 minutes at 37 °C under saturating conditions (10 μg/ml), washed and subsequently stained with FITC-conjugated goat anti-human secondary antibodies (Jackson Immunoresearch Lab. Inc., West Grove, PA). HIV-infected PBLs were taken for the analysis on day 8–11 after infection. All procedures were performed in TSM (20 mM Tris–HCl pH 8.0, 150 mM NaCl, 1 mM CaCl_2_, 1 mM MgCl_2_) buffer containing 2 % bovine serum albumin (BSA). In DC-SIGN blocking experiments, DC-SIGN-Fc was preincubated for 10 min at room temperature either with mannan (50 μg/ml) or EGTA (10 mM). Signals were acquired on a flow cytometer (FACSCalibur; BD Biosciences) and analyzed by CellQuest Software.

### ELISA

Samples after anion-exchange chromatography were directly coated onto ELISA plates (NUNC maxisorp, Nalge Nunc Int., Rochester, NY) in 0.2 M sodium carbonate buffer pH 9.2 for overnight at 4 °C followed by incubation in TSM buffer containing 1 % BSA for 30 min at 37 °C. DC-SIGN-Fc (ICAM-3-Fc as a negative control) (2 μg/ml) was added and incubated in TSM 1 % BSA 1 h at room temperature. Unbound DC-SIGN-Fc was washed away and the binding was determined using a peroxidase-conjugated goat anti-human Fc antibody (Jackson Immunoresearch Lab. Inc.) that was incubated for 30 min at room temperature. The analysis of the fractions for the presence of CD56 was performed with anti-CD56 mAbs (clone NCAM 16.2, BD Biosciences) as described above for DC-SIGN. The binding was determined using a peroxidase-conjugated rabbit anti-mouse IgG2b antibody (Zymed Laboratories, Inc.).

In the CD56 glycosylation ELISA analysis the anti-CD56 mAbs (clone NCAM 16.2, BD Biosciences, mouse Ig2b) mAbs were coated (5 μg/ml) and blocked with BSA as described above. PHA activated PBLs were lysed in NP-40 containing buffer for immuneprecipitation containing protease inhibitors (100 mln/ml) and then added and removed four times following 30 min incubation at RT with the coated anti-CD56 mAbs. The captured CD56 was analyzed with: a) anti-Lewis B, X or Y monoclonal antibodies (all mouse IgM, 3 μg/ml) following by the incubation with secondary anti-mouse IgM-peroxidase conjugate (Zymed Laboratories, Inc.); b) DC-SIGN-Fc as described above.

All ELISA experiments were performed in triplicate and several times.

### Immunoprecipitation and immunoblot analysis

PBLs after stimulation with PHA/IL-2 were cultured for 10–14 days in the presence of IL-2. To isolate cell surface CD56, preincubation of PBLs with anti-CD56 mAb (clone B159, BD) followed by washing was performed before the cell lysis. Cells were lysed in the buffer (1 % Triton-X–100, 10 mM TEA pH 8.2, 150 mM NaCl, 1 mM CaCl_2_, 1 mM MgCl_2_) containing a cocktail of EDTA-free protease inhibitors (Roche Diagnostics) and immune complexes were precipitated from the lysate with protA beads (CL-4B, Pharmacia). Immunoprecipitates were reduced in sample buffer (containing 4 % SDS and 5 % β-mercapto-ethanol), heated for 5 min at 95 °C, run on a 7 % polyacrylamide gel (SDS-PAGE) and transferred onto nitrocellulose membrane. Nitrocellulose membranes were incubated in blocking buffer (tris-buffered saline containing 0.1 % Tween 20 and 4 % BSA) and incubated overnight at 4 °C with the specified primary antibody or DC-SIGN-Fc diluted according to the manufacturer. Membranes were incubated with appropriate horseradish peroxidase conjugated secondary antibodies for 2 h and developed using the enhanced chemiluminescence detection system (Amersham, Buckinghamshire, UK).

Alternatively, the PBLs were surface biotinylated with sulfo-NHS-biotin (Pierce, Rockford, IL), and subsequently lysed. CD56 and DC-SIGN ligands were immune precipitated from lysates in specific buffers. All following procedures were carried out as described above but the membranes were stained with peroxidase-conjugated streptavidin (Vector, Burlingame, CA).

### Cytotoxicity assay

The lactate dehydrogenase (LDH)-based CytoTox 96 Assay (Promega) was used to determine *in vitro* cytotoxicity according to the manufacturer’s instructions. Briefly, the culture medium was changed to Opti-MEM supplied with 3 % heat-inactivated FCS for experimental cells immediately before the experiment following the extensive washing to avoid contamination with LDH from FCS. 4 × 10^4^ DCs (target cells)/sample, in quadruplicate were co-cultured with 2 × 10^5^ isolated CD56^pos^ cells (min 90 % purity). Alternatively: 1) DCs were preincubated with anti-DC-SIGN mAbs (to 30 μg/ml) for 30 min and cytotoxicity was performed in the medium containing these antibodies; 2) CD56^pos^ cells were preincubated for 4 h with the C3d peptide blocking NCAM homotypic interaction. After 4 h of incubation for each different experimental condition, released LDH into the culture supernatants was measured with a 30-min coupled enzymatic assay, which results in the conversion of a tetrazolium salt into a red formazan product that is read at 490 nm in an automated plate reader (Bio-Rad).

### Flow cytometry

Analytical flow cytometry was performed on FACS calibur (BD Pharmingen). Data analysis and graphics were acquired using the WinMDI 2.1 software package (http://facs.scripps.edu/software.html).

### Anion exchange chromatography (AEX)

Activated and cultured in the presence of IL-2 PBLs were washed with PBS and incubated with anti-CD56 mAbs (clone B159, BD Biosciences). Cells were lysed in the presence of 1 % NP-40 and cell surface CD56 was immuneprecipitated with protA beads (CL-4B, Pharmacia, Uppsala, Sweden). Immune precipitated complexes after washing were freed from the beads using 20× volume 0.1 M glycin-HCL buffer (pH 2.6) for 3 min RT with shaking. Beads were spinned down with 7000 g for 3 minutes. The supernatant pH was neutralized by adding 0.4 volume of 1 M Trsi-HCl (pH 7.5). Polysialilated CD56 was separated from weakly non-sialylated CD56 by means of anion exchange chromatography. AEX was carried out on a Surveyor LC system (Thermofinnigan) equipped with a strong anion exchange column (ProSphere polymeric SAX column, 75 × 7.5 mm, 1000A, 10 μ) and a Photo Diode Array detector. Separations were carried out using linear gradient from 0 to 0.5 M Ammonium Carbonate in MilliQ (freshly prepared) in 30 minutes at a flow-rate of 1 ml/min. Fraction of 1 ml were collected and concentrated in a speedvac.

### Statistical analysis

Significance was determined with unpaired *t* test (two ailed) and indicated in figures with stars. *, *p* ≤ 0.05; **, *p* ≤ 0.005; ***, *p* ≤ 0.0005. Data are presented as mean +/− SD (error bars).

## Abbreviations

AEX: anion exchange chromatography
BSA: bovine serum albumin
DC-SIGN: dendritic cell-specific intercellular adhesion molecule-3-grabbing non-integrin
DC-SIGN-L: DC-SIGN ligand
HIV-1: human immunodeficiency virus type 1
ICAM-3: intercellular adhesion molecule-3
iDCs and mDCs: immature and mature dendritic cells
Le^Y^: Lewis Y
MHC: major histocompatibility complex
NCAM: neural cell adhesion molecule CD56
NK: natural killer
PHA: phytohaemagglutinin
PSA: polysialic acid
